# Promoting Health Equity Through Partnerships

**DOI:** 10.1093/geroni/igab046.972

**Published:** 2021-12-17

**Authors:** Karon Phillips

**Affiliations:** Trust for America's Health, Trust for America's Health, District of Columbia, United States

## Abstract

Funded by The John A. Hartford Foundation, Trust for America’s Health’s (TFAH) Healthy Aging initiative has supported states as they develop Age-Friendly Public Health Systems (AFPHS). The goal of this national initiative is to make healthy aging a core function of state and local public health departments. Through this initiative, TFAH is working directly with states as they work to improve the health of older adults, with a particular focus on health equity. Given the increased prevalence of health disparities, prioritizing health equity has become important for many organizations. Through new partnerships and collaboration with aging services providers and health care systems, public health departments have developed innovative ways to improve the health and well-being of older adults from racial/ethnically diverse backgrounds. Areas of collaboration between the public health and aging sectors include sharing data on older adult health and working together to address social isolation.

